# Peptide Blocking CTLA-4 and B7-1 Interaction

**DOI:** 10.3390/molecules26020253

**Published:** 2021-01-06

**Authors:** Stepan V. Podlesnykh, Kristina E. Abramova, Anastasia Gordeeva, Andrei I. Khlebnikov, Andrei I. Chapoval

**Affiliations:** 1 Russian-American Anti-Cancer Center, Altai State University, 61 Lenin St., 656049 Barnaul, Russia; step-uch@mail.ru (S.V.P.); abramova.k.e@gmail.com (K.E.A.); anastasia.17.98@mail.ru (A.G.); 2 Kizhner Research Center, National Research Tomsk Polytechnic University, 30 Lenin St., 634050 Tomsk, Russia; aikhl@chem.org.ru; 3 Center for Innovations in Medicine, Biodesign Institute, Arizona State University, 1001 S. McAllister Ave., Tempe, AZ 85281, USA

**Keywords:** peptides, immune checkpoints, peptide microarray, cancer, immunotherapy

## Abstract

Discovery of the B7 family immune checkpoints such as CTLA-4 (CD152), PD-1 (CD279), as well as their ligands B7-1 (CD80), B7-2 (CD86), B7-H1 (PD-L1, CD274), and B7-DC (PD-L2, CD273), has opened new possibilities for cancer immunotherapy using monoclonal antibodies (mAb). The blockade of inhibitory receptors (CTLA-4 and PD-1) with specific mAb results in the activation of cancer patients’ T lymphocytes and tumor rejection. However, the use of mAb in clinics has several limitations including side effects and cost of treatment. The development of new low-molecular compounds that block immune checkpoints’ functional activity can help to overcome some of these limitations. In this paper, we describe a synthetic peptide (p344) containing 14 amino acids that specifically interact with CTLA-4 protein. A 3D computer model suggests that this peptide binds to the ^99^MYPPPY^104^ loop of CTLA-4 protein and potentially blocks the contact of CTLA-4 receptor with B7-1 ligand. Experimental data confirm the peptide-specific interaction with CTLA-4 and its ability to partially block CTLA-4/B7-1 binding. The identified synthetic peptide can be used for the development of novel immune checkpoint inhibitors that can block CTLA-4 functional activity for cancer immunotherapy.

## 1. Introduction

Receptors for the B7 family ligands expressed on the surface of T lymphocytes can deliver both stimulating and inhibitory signals that regulate the immune response [1,2]. Inhibitory receptor CTLA-4 (CD152) interact with ligands B7-1 (CD80) and B7-2 (CD86), while PD-1 (CD279) receptor binds B7-H1 (PD-L1, CD274) and B7-DC (PD-L2, CD273). These molecules are also termed as immunological checkpoints [1,2]. Injections of monoclonal antibodies (mAb) blocking immunological checkpoints, such as CTLA-4, PD-1, and PD-L1, enhance antitumor immunity and lead to tumor rejection [3,4,5,6]. However, the side effects and cost of manufacturing mAb for therapy limit their wider clinical use. Low molecular weight agents that block immunological checkpoints can overcome some problems associated with the use of mAb for cancer therapy [7,8].

Several groups are developing low molecular weight agents such as aptamers, small molecules, binding parts of antibodies, and peptides for disruption of CTLA-4 binding to its ligands [9,10,11,12,13]. Peptides for the therapy of various diseases are gaining more interest because of cell-free low-cost production, optimal half-life in circulation, ability to specifically bind to other proteins, and higher tissue penetration [14]. In this work, we introduce a peptide (p344), which can specifically bind the CTLA-4 molecule. This peptide was identified using microarrays containing 330034 peptides with random amino acid sequences [15,16,17]. It appears that the identified peptide interacts with the ^99^MYPPPY^104^ amino acid sequence loop of the CTLA-4 molecule which is responsible for the binding of CTLA-4 with B7-1 [18]. In this work, we confirmed the specificity of p344 peptide interaction with the CTLA-4 protein using ELISA. p344 peptide presented in this work is one of the leading candidates for the development of a low-molecular agent that blocks the interaction of CTLA-4 with its ligands. This peptide can be potentially used for immunotherapy of cancer and other immunologically dependent diseases.

## 2. Results and Discussion

Earlier, using peptide microarrays, we identified >350 peptides that specifically interact with the recombinant fusion proteins B7-1Fc, B7-2Fc, and CTLA-4Fc [15]. Nineteen peptides out of 330034 peptides presented on microarrays, specifically interacted with recombinant CTLA-4Fc protein. We synthesized eight peptides that showed maximal interaction with CTLA-4Fc. Among synthesized peptides, p344 peptide with amino acid sequence ARHPSWYRPFEGCG demonstrated reproducible results in a series of experiments and was selected for further analysis. This synthetic peptide (Mw = 1.66 kDa) contains 14 amino acid residues, the calculated values of the GRAVY index = −1.114, characterize this peptide as hydrophilic. Control peptide p333 (amino acid sequence EGLNRPSGGCG), which does not interact with CTLA-4Fc, has similar characteristics.

**Figure 1:**
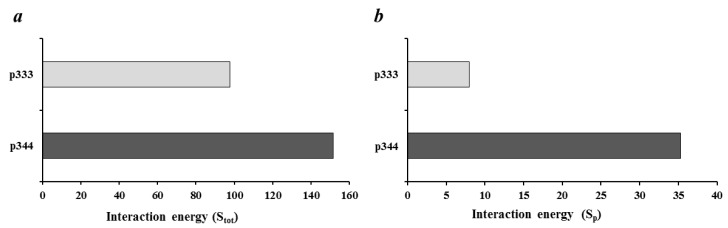
Assessment calculated interaction energy of p344 peptide with CTLA-4. (**a**) Total Docking Score of peptide interaction with the whole extracellular portion of CTLA-4 (S_tot_); (**b**) Contribution to the Docking Score of peptide interaction with ^99^MYPPPY^104^ loop of CTLA-4 molecule (S_p_).

We used a computational approach implemented on the CABS-dock server [19], which is intended for docking peptides without knowing the binding site on the protein macromolecule. In this case, the conformational mobility of the peptide and partial mobility of the protein are considered [19], which allows docking without special preparation (pre-optimization) of the crystal structure of the macromolecule. First, we found the optimal docking poses and calculated interaction energy of CTLA-4 with peptide p344 and control p333. When we analyzed the calculated interaction energy of p344 and control p333 peptide with the whole CTLA-4 extracellular domain (S_tot_), both peptides showed similar values (Figure 1a). However, when we compared the interaction energy of these peptides with the ^99^MYPPPY^104^ loop, p344 peptides showed significantly higher values (S_p_), suggesting that the p344 peptide position on the CTLA-4 molecule is energetically optimal near the ^99^MYPPPY^104^ loop (Figure 1b). It is known that the ^99^MYPPPY^104^ loop of CTLA-4 is responsible for the binding of CTLA-4 with B7-1 [18].

**Figure 2:**
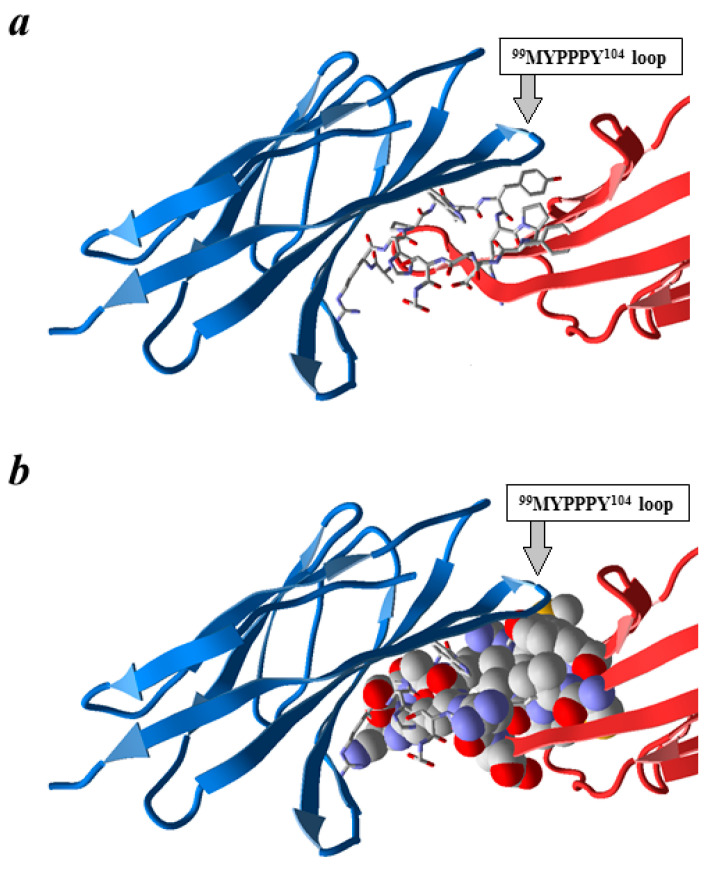
Model of p344 peptide interaction with CTLA-4 and B7-1 proteins. Using the CABS-dock server, p344 was docked to CTLA-4 protein (blue ribbon). Next, the B7-1 ligand (red ribbon) was embedded in the CTLA-4/p344 complex as in PDB 1I8L. p344 shown as sticks (**a**,**b**); the residues of B7-1 which are in clash with the docked peptide are shown as spheres (**b**).

Next, we analyzed the obtained 3D model of p344 peptide bound to the extracellular portion of CTLA-4. Figure 2 shows the results of molecular docking for p344 peptide to the extracellular part of the CTLA-4 molecule. When the B7-1 ligand was embedded in the CTLA-4/p344 docking complex in the orientation adopted in the 1I8L structure, a noticeable superposition of B7-1 on the docked peptide was observed (Figure 2a,b). According to our calculations, 15 amino acid residues of B7-1 protein are located within 1 Å of the p344 peptide on the CTLA-4 molecule, so they are considered to be in clash with the peptide and therefore prevent CTLA-4 binding to B7-1 (Figure 2b). Thus, it is conceivable that the p344 peptide can cause a blockade of CTLA-4 and B7-1 interaction.

Further analysis of the modeled CTLA-4/p344 complex showed that the following eleven residues of CTLA-4 are located within 3 Å from the docked peptide: *Glu33*, Val34, *Arg35*, Thr37, Glu48, Ala51, *Thr53*, *Leu63*, Glu97, *Met99*, and *Tyr100*. Among them, Glu33, Thr37, and Thr53 form hydrogen bonds with residues Tyr7, Ser13, and Pro4 of the peptide, respectively. Residues Val34, Glu48, and Ala51 of CTLA-4 participate in attractive van der Waals interactions with p344 peptide (evaluated by “Energy inspector” tool of MVD software). Measurements on CTLA-4/B7-1 complex (PDB 1I8L) gave the following list of thirteen CTLA-4 residues in the vicinity of 3 Å from B7-1 ligand: *Glu33*, *Arg35*, *Thr53*, *Leu63*, Asp65, Lys95, *Met99*, *Tyr100*, Pro102, Pro103, Tyr104, Tyr105, and Leu106. It is seen that both lists contain common residues of CTLA-4 (shown in italic), indicating that the formation of CTLA-4/p344 complex prevents an effective interaction between CTLA-4 and B7-1. It should be noted that Met99 and Tyr104 residues of CTLA-4 are involved in H-bonding with Tyr31 and Lys36 of the B7-1 ligand, respectively. Additionally, Arg29, Lys89, and Asp90 residues of B7-1 form salt bridges with Glu33, Asp65, and Arg35 of CTLA-4 protein. These anchoring interactions must be significantly affected by the p344 peptide.

**Figure 3:**
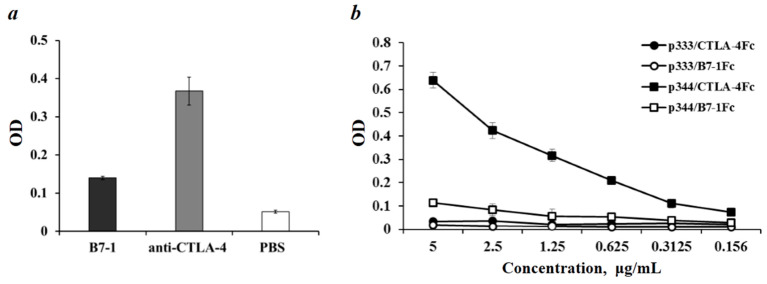
Interaction of CTLA-4Fc with ligand, antibodies, and synthetic peptides. (**a**) CTLA-4Fc fusion protein 5 μg/mL was added to the wells with immobilized recombinant B7-1 protein (8 μg/mL) and anti-CTLA-4 mAb (8 μg/mL). Protein-free wells (PBS) were used as a negative control. (**b**) CTLA-4Fc or B7-1Fc at indicated concentrations were added to the wells with immobilized synthetic peptides p344 or p333 (2 μg/mL). Binding of fusion proteins to the wells was determined using HRP conjugated mAb against the Fc part of human IgG.

Next, we investigated by ELISA whether CTLA-4Fc can bind synthetic peptide p344. First, we confirm the ability of CTLA-4Fc fusion protein to bind B7-1 recombinant protein and mAb against human CTLA-4. The data presented in Figure 3a shows that the recombinant CTLA-4Fc chimeric protein specifically interacts with the recombinant B7-1 protein and antibodies against human CTLA-4 protein. The higher level of interaction of the recombinant CTLA-4Fc protein with the anti-CTLA-4 mAb can be explained by the higher affinity of antibodies compared to the B7-1 protein.

To verify the specificity of CTLA-4Fc interaction with synthetic peptides we incubated CTLA-4Fc and B7-1Fc with immobilized p344 or p333 peptides. Data presented in Figure 3b show that B7-1Fc did not bind p344 and p333 peptides. In contrast, CTLA-4Fc binds p344 in a dose-dependent manner (Figure 3b). No significant CTLA-4 interaction with control p333 peptide was observed. It appears that the Fc part of the CTLA-4Fc fusion protein did not participate in p344 peptide binding, since B7-1Fc protein containing the same amino acid sequence Fc did not bind p344. Therefore, the extracellular portion of the CTLA-4 molecule specifically binds the p344 peptide.

**Figure 4:**
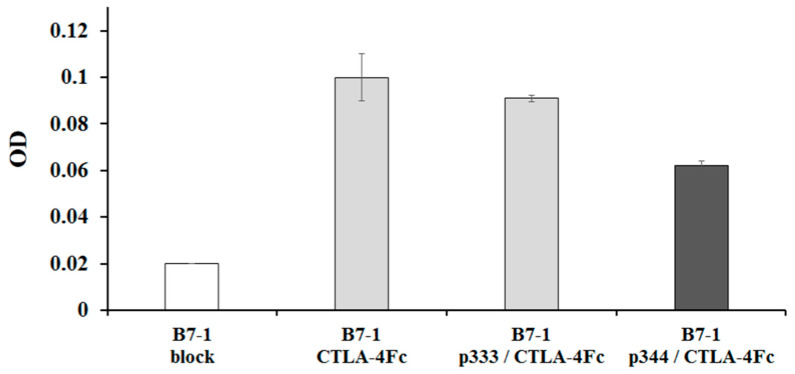
Blocking CTLA-4 and B7-1 interaction with p344 peptide. CTLA-4Fc (5 μg/mL) was incubated with p344 or p333 peptide (8 μg/mL) for 2 h and added to the wells of a 96-well plate with immobilized recombinant B7-1 protein (8 μg/mL). The interaction of CTLA-4Fc with B7-1 was determined using HRP conjugated mAbs against the Fc part of human IgG. One of four independent experiments is shown.

The next step in this study was to evaluate the ability of synthetic peptides to block the binding of CTLA-4 to its natural ligand B7-1 (Figure 4). In these experiments, CTLA-4Fc was preincubated with either p344 or control p333 peptide and added to the wells with immobilized recombinant B7-1 protein (without Fc fragment). Data indicate that the addition of CTLA-4Fc to immobilized B7-1 increased optical density from 0.02 (background) to 0.105, similar data presented in Figure 3а. This result indicates that CTLA-4Fc bind to B7-1. Preincubation of CTLA-4Fc with p333 did not notably change CTLA-4Fc and B7-1 binding. However, incubation of CTLA-4Fc in the presence of p344 decreased the binding of CTLA-4 to B7-1 by 42%. Thus, these results indicate that the p344 peptide, which specifically interacts with CTLA-4, can partially block CTLA-4 and B7-1 interactions.

Thus, during this work, a peptide interacting with the CTLA-4 molecule was identified and characterized. Using molecular modeling, we have shown that p344 peptide interacts with ^99^MYPPPY^104^ loop of CTLA-4, which is responsible for binding CTLA-4 to the B7-1 ligand. The experimental data obtained by ELISA confirmed modeling results regarding specificity CTLA-4 and p344 peptide interaction. Moreover, we have shown that p344 partially blocks the interaction of CTLA-4 with B7-1, which also confirms p344 binding near the MYPPPY motif.

The p344 peptide presented in this work is a leading molecule for the development of immunostimulatory drugs that can regulate/prevent binding of ligands B7-1 (CD80) (and possibly B7-2 (CD86)) to the inhibitory CTLA-4 receptor and thereby may enhance the immune response. However, immunomodulatory properties of p344 peptide require validation in vitro and in vivo, which will be reported in upcoming publications.

Synthetic peptides capable of blocking immunological checkpoint can be used as a substitute of mAb for immunotherapy of autoimmune diseases and cancer. Peptides compare to mAb, have several advantages; including small size, which provides more efficient penetration into tissues or tumors; low immunogenicity, which allows an unlimited number of injections; relatively simple chemical synthesis, which reduces the cost of the drug. The strategy described in this paper can be used to search for peptides interacting with other molecules that regulate the immune response, for example, PD-1, PD-L1, and PD-L2, B7-H3 (CD276), B7-H4, B7-H5 (VISTA) other.

## 3. Materials and Methods

Peptides used in this work were identified using a microarray containing 330034 peptides with random amino acid sequences, according to the protocol described earlier [15]. Briefly, recombinant fusion protein CTLA-4Fc, containing Fc portion of human IgG, was applied on peptide microarrays, and interaction with peptides was detected using fluorescence-labeled mAb against human IgG. For this work, we used peptide p344 with amino acid sequence ARHPSWYRPFEGCG which interacted with the CTLA-4Fc molecule. Another peptide p333 (amino acid sequence EGLNRPSGGCG) did not bind CTLA-4 in the experiments with microarrays and was used in this work as a negative control.

For 3D modeling, the structure of the B7-1/CTLA-4 complex was downloaded from the Protein Data Bank (code 1I8L). The substructure containing an extracellular portion of CTLA-4 protein was exported from the downloaded file and sent to the CABS-dock server (protein-peptide docking software http://biocomp.chem.uw.edu.pl/CABSdock/) [19]. When performing the simulation, the strategy of “blind” docking was used without specifying the binding site on the CTLA-4 molecule: only the protein structure and amino acid sequence of the peptide were uploaded to the server; the optional search settings were not applied. The best conformation of the p344 peptide in complex with the CTLA-4 molecule, obtained as an output from the CABS-dock server, was imported into the Molegro Virtual Docker (MVD) program (Molegro ApS, Denmark) for further analysis and visualization. The Docking Score value reflecting the degree of interaction of peptides with an extracellular part of CTLA-4 molecule (S_tot_), as well as the contribution to this value made only by the ^99^MYPPPY^104^ loop amino acid sequence (S_p_), was estimated using the Ligand Energy Inspector tool included in the MVD program. When calculating S_tot_ and S_p_, the MolDock score function was used [20].

In the experimental work, we used recombinant fusion proteins CTLA-4Fc and B7-1Fc, containing Fc portion of human IgG, purchased from R&D Systems (USA). Phosphate-buffered saline (PBS) (Biolot, Russia), Tween-20 (Helicon, Russia), and bovine serum albumin (BSA) (Amresco, USA) were used for the preparation of basic working solutions. High-purity pyrogen-free deionized water was obtained using the UVOI-MF-1812 water treatment system (Akvalab, Russia).

The peptides p344 (ARHPSWYRPFEGCG) and p333 (EGLNRPSGGCG) were synthesized at OOO “Alfa-Organika” (Russia) using the methods of classical peptide synthesis in solution. The resulting lyophilized peptides were dissolved in PBS (pH 7.3) and stored at 4 °C.

The binding specificity of peptides to recombinant protein molecules was determined by ELISA. Before peptide coating, 96-well plates (Corning, USA) were treated with glutaraldehyde (0.125%) 200 μL/well for 2 h at room temperature. After washing with PBST (PBS + 0.05% Tween-20) and drying the plates synthetic peptides 100 μL/well at 2 μg/mL in PBS (pH 9.5) were added to the wells and incubated for 3 h at 37 °C in a shaker. After peptide coating 200 μL of blocking solution containing 2% BSA and 0.05% Tween-20 in PBS was added to each well and plates were incubated for 2 h at room temperature. After washing different concentrations of CTLA-4Fc and B7-1Fc were added into the wells in triplicates 100 μL/well and plates were incubated for 1 h at 37 °C. After washing 100 μL of anti-human IgG antibody conjugated with HRP (1:10.000 dilution) was added to the wells and incubated for 1 h at room temperature. Plates were washed six times with PBST solution and TMB substrate was added (50 μL/well). Plates were kept in the dark at room temperature for 1–5 min. The reaction was stopped by adding 50 μL of 1 M Н_2_SO_4_ to each well. The optical density of the reaction was recorded at wavelength 450 nm using an iMark photometer (Bio-Rad, Hercules, CA, USA).

The binding of CTLA-4Fc to B7-1 and mouse mAb against human CTLA-4 was also assessed by ELISA. Recombinant protein B7-1 without Fc or mouse mAb against human CTLA-4 (R&D Systems) were coated on the plates at 8 μg/mL wells. CTLA-4Fc was added to the wells at 5 μg/mL. Binding of CTLA-4Fc was detected with anti-human IgG HRP.

The blocking activity of peptides was analyzed in similar experiments. CTLA-4Fc (5 μg/mL) was incubated with p344 or p333 (8 μg/mL) peptides and added to the wells coated with 8 μg/mL B7-1 without Fc. Binding of CTLA-4Fc was detected with anti-human IgG HRP, as described above.

## Data Availability

The data presented in this study are available on request from the corresponding author.

## Sample Availability

Samples of selected compounds are available from the authors.
